# A Rapid Fluorescence Quenching Assay for Total Levothyroxine Quantification in Pharmaceutical and Supratherapeutic Serum Samples

**DOI:** 10.1007/s10895-025-04510-9

**Published:** 2025-09-15

**Authors:** Enas T. Abdel-Salam, Zeinab M. Anwar, Ahmed Z. Ibrahim, Hend M. Musatfa

**Affiliations:** 1 Faculty of Science, Chemistry Department, Suez Canal University, Ismailia, Egypt; 2 Research Faculty of Science,Chemistry Department, El- Arish University, ElArish, Egypt

**Keywords:** Levothyroxine, Thyroid, UV, Absorbance, T4, Spectrofluorometry, Hypothyroidism, Drug, Fluorescence quenching, Binding constant

## Abstract

A rapid, cost-effective spectrofluorometric method was developed for levothyroxine (T4) detection using fluorescein isothiocyanate (FITC) as a fluorescent probe. The method exploits FITC fluorescence quenching by T4 in Tris/phosphate buffers (pH 7.4),with linear ranges of 8.00 × 10⁻⁶ – 6.40 × 10⁻⁵ mol/L and detection limits of 9.60 × 10⁻⁶ mol/L (Tris) and 8.80 × 10⁻⁶ mol/L (phosphate). Stern-Volmer analysis confirmed dynamic quenching dominated by electrostatic interactions. The method demonstrated 102–104% recovery in spiked human serum and minimal interference from common drugs (Ciprofloxacin, Alendronate) and ions (Fe³⁺, Al³⁺). Compared to HPLC and kinetic methods, this approach offers simplicity, speed, and accessibility for routine pharmaceutical/clinical analysis.

**Supplementary Information:**

The online version contains supplementary material available at 10.1007/s10895-025-04510-9.

## Introduction

Levothyroxine (L-thyroxine, T4) is a synthetic thyroid hormone frequently administered for the management of hypothyroidism and associated endocrine diseases. It replicates the physiological function of endogenous thyroxine produced by the thyroid gland, which is essential for regulating metabolic processes, oxygen utilisation, and cellular differentiation. The absorption and bioavailability can be markedly influenced by interactions with co-administered compounds, including metal ions such as calcium, iron, and aluminium, as well as pharmaceutical medicines like ciprofloxacin and alendronate. The nature of these interactions, whether chemical or pharmacokinetic, is under investigation.

A multitude of analytical techniques has been established for the quantification of levothyroxine in pharmaceutical formulations and biological matrices. High-performance liquid chromatography (HPLC) is the predominant technology utilised, frequently combined with photodiode array detection [1], chiral stationary phases [2], or tandem mass spectrometry to enhance specificity and sensitivity [3]. Kinetic techniques utilising catalytic oxidation [4], electrochemical sensors [5], and inductively coupled plasma mass spectrometry (ICP-MS) [6] have been investigated. Although these procedures provide elevated sensitivity and selectivity, they frequently need substantial resources, including advanced gear, intricate sample preparation, and highly skilled staff.

### Comprehensive Survey of Current T_4_ Assays

Over the past two decades, reversed-phase high-performance liquid chromatography (HPLC) has remained the primary technique for routine Levothyroxine (T₄) analysis. Early studies utilizing quinine-derived chiral columns achieved baseline separation of d-/l-T₄ and T₃, with quantification limits below 0.5 µg/mL in pharmaceutical tablets [7]. Subsequent advancements extended the linear range down to 0.08 µg/mL for dissolution testing, though at the cost of prolonged analysis times (35–45 min) and large sample injection volumes [8, 9].

More recently, liquid chromatography–tandem mass spectrometry (LC-MS/MS) has emerged as the gold standard for ultra-trace quantification of Levothyroxine in biological samples. These methods can achieve detection limits as low as 0.3 ng/mL in serum, following ultrafiltration and isotope dilution, but require high-end instrumentation, skilled operators, and extensive sample preparation [10–12]. Direct elemental detection by inductively coupled plasma mass spectrometry (ICP-MS) also achieves high sensitivity (~ 0.1 ng/mL) via iodine detection, yet its widespread adoption remains limited by the need for expensive equipment and specialized sample treatments [13, 14].

### Non-Chromatographic Alternatives

Catalytic–kinetic spectrophotometry has also been applied for Levothyroxine determination, particularly using Mn(III)/As(III) redox systems. These methods can detect T₄ at low-nanomolar concentrations (2.7 nmol/L), but involve hot-acid reagents, longer reaction times, and elevated temperatures, limiting routine use [15, 16]. Electrochemical approaches—often using modified glassy carbon or carbon-paste electrodes—offer inexpensive alternatives with detection limits reaching the sub-micromolar range, though their application is challenged by electrode fouling, surface effects, and relatively narrow linear ranges [14, 17].

### Emerging Fluorescence-Based Methods

Fluorescence-based detection techniques are comparatively underexplored for Levothyroxine. Recent innovations include aptamer-based fluorescence sensors and ratiometric fluorescent probes targeting thyroid hormones and related iodinated species [18, 19]. While offering promising sensitivity and selectivity, these systems typically require advanced aptamer engineering or specialized probe synthesis, which may limit accessibility for routine laboratory analysis.

### Positioning of the Present Work

The FITC-based fluorescence quenching assay presented in this work provides a practical middle ground between analytical simplicity, cost-efficiency, and operational speed. While its detection limit (9.6 µmol/L in Tris; 8.8 µmol/L in phosphate buffer) is modest compared to LC-MS/MS or ICP-MS protocols, the assay completes within six minutes at room temperature, requires only a bench-top fluorimeter and non-hazardous buffers, and operates directly at physiological pH without derivatization. These features make it particularly suitable for high-throughput pharmaceutical quality control or monitoring of supratherapeutic serum levels—applications where ultra-trace sensitivity is unnecessary and cost-per-sample is a critical factor. Furthermore, it fills a methodological gap for laboratories seeking rapid, reagent-free screening techniques [9].

**Table:** 

Method (ref.)	LOD (µg L⁻¹)	Linear range (µg L⁻¹)	Assay time (min)	Instrument cost	Sample prep	Distinct merit
LC-MS/MS [10]	0.01	0.03–8.0	45	Very high	Ultrafiltration	Gold-standard selectivity
ICP-MS [13]	0.03 (as I⁻)	0.1–50	30	Very high	Dilution	Direct iodine read-out
Gradient HPLC–UV [8]	0.08	0.08–0.8	35	High	Filtration	Tablet dissolution profiling
Catalytic kinetic spectrophotometry [15]	0.67	2.2–9.9	25	Low	Acidification	Sub-nmol sensitivity without optics
This work – FITC quench	9.6	8–64	6	Very low	Simple dilution	Fast, room-temperature, no hazardous reagents

Fluorescence spectroscopy has emerged as a potential alternative to meet the demand for a more straightforward and accessible technique. It provides swift, precise, and non-invasive analysis. Fluorescein isothiocyanate (FITC) is a widely utilised fluorophore, characterised by its robust absorption (~ 493 nm) and emission (~ 516 nm) within the visible spectrum. FITC exhibits a high reactivity with primary amines, forming complexes that modify its fluorescence characteristics, rendering it appropriate for probe-based sensing applications. Fluorescence spectroscopy has emerged as a powerful analytical tool for detecting trace amounts of biologically and pharmaceutically important compounds due to its high sensitivity, selectivity, and simplicity. In particular, the use of organic fluorescent dyes, such as fluorescein isothiocyanate (FITC), enables precise quantification of analytes through fluorescence quenching or enhancement mechanisms. Ion-pair complex formation between target molecules and dyes has proven especially effective in improving detection limits and enhancing analytical performance. Several recent studies have reported successful applications of this approach for various compounds, demonstrating its versatility and reliability in analytical chemistry. Moreover, fluorescence-based detection methods offer significant advantages in terms of environmental sustainability and operational simplicity. A number of recent reports have demonstrated the utility of fluorescent probes for trace-level drug quantification in biological matrices while maintaining green chemistry principles [20–22].

For example, fluorescence-based assays involving ion-paired complexes and quenching mechanisms have been effectively applied for the detection of antihypertensive and anti-inflammatory drugs [23], heavy metal ions [24], and bioactive molecules using FITC and related dyes [25]. Additionally, a recent study on levothyroxine quantification using fluorescence probes demonstrated the practical potential of fluorescence quenching and ion-pair formation strategies in routine analysis [26]. These studies collectively highlight the expanding role of fluorescence techniques in pharmaceutical analysis and provide valuable insights for developing sensitive methods for levothyroxine determination.

The fluorescence quenching resulting from interactions with analytes can be quantitatively assessed using the Stern–Volmer and Lineweaver–Burk models to get binding and quenching constants.

This paper introduces a spectrofluorometric technique for quantifying levothyroxine utilising FITC as a fluorescent probe. The methodology entails the examination of fluorescence quenching in Tris and phosphate buffer systems (pH 7.4), assessing the quenching processes, thermodynamic characteristics, and binding constants. The interference from frequently encountered metal ions (Fe³⁺, Al³⁺, Ca²⁺) and pharmaceuticals (ciprofloxacin, alendronate) was evaluated, and the methodology was effectively utilised on human serum samples using standard addition. This method’s simplicity, cost-effectiveness, and adequate sensitivity provide it a viable instrument for regular pharmaceutical and clinical study of levothyroxine.

#### Chemistry and Pharmacology of Levothyroxine

##### Thyroxine Function

**Fig. 1:**
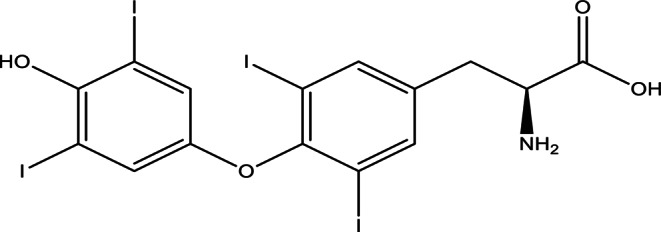
Levothyroxine (T4)

**Fig. 2:**
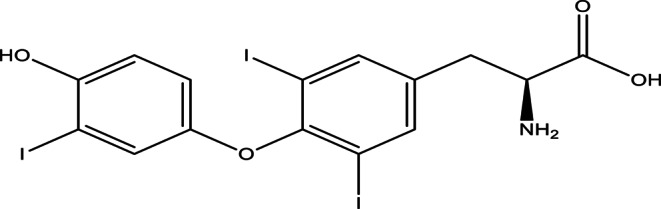
Triiodothyronine(T3)

The thyroid plays a key role in regulating metabolism for the rest of the body. The hormones excreted by the Thyroid are thyroxin (T4), tri-iodothyronine (T3), and calcitonin. The structures of these secreted hormones are depicted in Figs. 1 and 2.

Levothyroxine (L-T4), a synthetic thyroid hormone, is essential for the management of hypothyroidism and the regulation of metabolic processes. It comprises three ionisable groups (carboxyl, phenolic, and amino) -as shown in Fig. 1- that affect its solubility and bioavailability. In aquatic environments, L-T4 occurs as a zwitterion or anion contingent upon pH, with solubility enhancing above pH 7. The interactions with concomitantly administered medications (e.g., ciprofloxacin, alendronate) and metal ions (Fe³⁺, Ca²⁺, Al³⁺) can modify absorption, requiring dependable analytical techniques for measurement in pharmaceutical and biological matrices [1]. 

Thyroxine binding glubiolin (TBG) exhibits the greatest affinity for binding with thyroid hormone and is stimulated by oestrogen and thyroid hormone. Transthyretin, previously known as thyroxine binding pre-albumin (TBPA), is present in both serum and cerebrospinal fluid, exhibiting a somewhat lower affinity; serum albumin, while having a low affinity for thyroid hormone, is abundant in serum.

Table 1 Presents the reference normal ranges for thyroid hormones, TSH, and thyroxine binding globulin [3].

**Table 1:** Reference normal ranges for thyroid hormones, TSH and TGB

Test	Method	USC Reference Ranges
Total Thyroxine (TT4)	Roche Cobas	57-159 nmol/L (4.5-12.5 ug/dL
Total Triiodothyronine (T3)	Roche Cobas	1.2-2.8 nmol/L (80-180 ug/dL
Thyroid Hormone Binding Ratio (THBR)	Roche Cobas	0.72-1.24 (unitless)
Thyrotropin (TSH)	Roche Cobas	0.3-4.0 mIU/L
Thyroxine Binding Globulin (TBG)	Siemens Immulite	14.0-31.0 mg/L (14.0-31.0 μg/mL

High-performance liquid chromatography (HPLC) was employed alongside mass spectrometry and amperometric detection to identify and quantify the degradation products and impurities of photo-sensitive Na-thyroxine. Seven breakdown components were isolated using HPLC with amperometric detection. These compounds, primarily present as impurities, were discovered using an advanced liquid chromatography-mass spectrometry approach. The identical HPLC approach was employed to examine Na-thyroxine and its degradation products in various commercially available brands of Na-thyroxine medications.

##### Fluorescence Quenching Processes

Fluorescence quenching occurs via dynamic (collisional) or static (complex formation) mechanisms. Dynamic quenching follows the Stern-Volmer relationship in Eq. 1.

1 $${\mathrm F}_0/\mathrm F=1+{\mathrm K}_{\mathrm{SV}}\lbrack\mathrm Q\rbrack$$

Where K_SV_​reflects quenching efficiency.

Static quenching involves non-fluorescent complex formation also following Eq. 1.

Differentiation is achieved via lifetime measurements or temperature dependence: dynamic quenching increases with temperature, while static quenching decreases. The proposed method leverages FITC’s fluorescence quenching by L-T4, analyzed through Stern-Volmer and Lineweaver-Burk models to elucidate binding constants and mechanisms [5]. – [6].

Figure 2 shows types of fluorescence quenching.

**Fig. 3:**
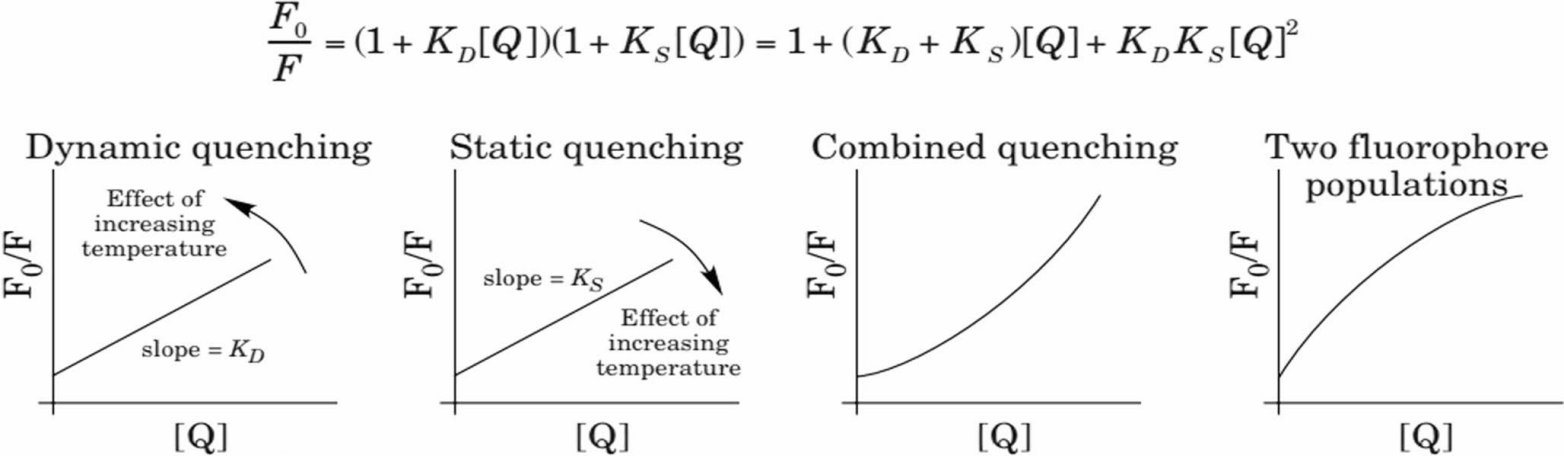
Quenching types

#### Literature Review on Analytical Estimation of Levothyroxine

**Gika et al.(2004) **developed a rapid reversed-phase HPLC method for the simultaneous separation and analysis of d- and l-thyroxine (d- and l-T4) and tri-iodothyronine (T3) utilising a quinine-derived chiral stationary phase, which was employed for the quantitative assessment of enantiomeric impurities in pharmaceutical formulations of levothyroxine. The impact of operational parameters has been examined for the optimisation of separation and to further understanding of the retention mechanism. The technique validation encompassed linearity, precision, and accuracy, demonstrating R.S.D. values below 3.3% for intra-assay precision and percent error between − 6% and + 2.1% for the specified validation samples, indicating satisfactory accuracy. Quantitation was conducted within the range of 0.5–500 µg/mL, with detection and quantitation limits below 0.1 and 0.5 µg/mL, respectively, for both analytes [7].

**Gika et al(2005)** explored the identification, isolation, and quantification of iodo amino acids, which are vital for biological research and the clinical diagnosis of thyroid gland diseases. A reversed-phase high-performance liquid chromatographic method was developed for the quantification of thyroid hormones and several of their primary metabolites, including 3,3’,5,5’-tetra-iodo-l-thyronine (l-thyroxine), 3,3’,5-tri-iodo-l-thyronine, 3,5-di-iodo-l-thyronine, l-thyronine, 3,5-di-iodo-l-tyrosine, 3-iodo-l-tyrosine, and l-tyrosine. Analysis was conducted using an Inertsil C18 Column employing photodiode-array detection, utilising a 25-minute gradient scale program of a binary mobile phase composed of a 0.1% aqueous solution.

Tri-fluoroacetic acid at pH 3 served as solvent A, while acetonitrile functioned as solvent B, with a flow rate of 1 mL/min. Quantification was conducted utilising theophylline as the internal standard. The approach was utilised on commercial medications and biological specimens (serum, urine, and tissue). Urine and serum samples devoid of drugs were spiked with known quantities of analyte standards and subjected to solid phase extraction to eliminate matrix interferences. C18 cartridges were employed, resulting in recoveries between 87.1% and 107.6% for serum samples, and between 92.1% and 98.7% for urine samples [27]. 

**Cu et al. (2007** demonstrated the use of an API-5000 tandem mass spectrometer, fitted with a Turbo Ion Spray source and a Shimadzu HPLC system, to conduct the study by isotope dilution employing a deuterium-labeled internal standard, T4-d5. Four hundred microlitres of human plasma/serum were subjected to filtration using a Centrifree YM-30 ultrafiltration device by centrifugation, after which 450 µL of an internal standard in methanol was incorporated into 150 µL of the ultrafiltrate for deproteinisation. Following centrifugation, 500 µL of supernatant was diluted with 400 µL of distilled deionised water, and a 650 µL aliquot was injected onto a C-18 column. Subsequent to washing, the switching valve was engaged, and the analytes were eluted from the column using a water/methanol gradient into the MS/MS apparatus. Quantification was conducted using multiple reaction-monitoring (MRM) analysis in the negative mode. The within-day and between-day coefficients of variation (CVs) were ≤ 9% for FT3 and ≤ 7% for FT4 across all tested concentrations. Accuracy varied from 95 to 105%. The 2.5th–97.5th percentiles for FT3 and FT4 were 0.09–0.4 ng/dL (1.4–6.2 pmol/L) and 0.8–2.1 ng/dL (10–26 pmol/L), respectively. The results exhibited only a moderate correlation with the immunoassays [10]. 

**Pastor et al.(2007)** investigated a novel, highly sensitive, and straightforward kinetic approach for the detection of thyroxine. The technique relied on the catalytic influence of thyroxine in the oxidation of As(III) by Mn(III) metaphosphate. The reaction kinetics were examined in the presence of orthophosphoric acid. The reaction rate was monitored spectrophotometrically at 516 nm. It was determined that orthophosphoric acid enhanced the reaction rate, but the magnitude of the non-catalytic reaction was negligible. A kinetic equation was proposed, and the apparent rate constant was determined. The relationship between reaction rate and temperature was examined, and the activation energy along with other kinetic parameters was ascertained. Thyroxine was quantified under optimal experimental circumstances within the range of 7.0 × 10^−9^ to 3.0 × 10^−8^ mol/L, exhibiting a relative standard deviation of up to 6.7% and a detection limit of 2.70 × 10^−9^ mol/L. With 0.08 mol/L chloride, the detection limit dropped to 6.60 × 10^−10^ mol/L. The proposed method was utilised for the quantification of thyroxine in tablets. The method’s accuracy was assessed through comparison with the HPLC method [15]. 

**Pabla et al. (2008)**, studied a simple, sensitive, and reproducible inductively coupled plasma mass spectrometry (ICP-MS).

Method for the direct quantification of levothyroxine (T4) through the examination of iodide concentration in aqueous solutions. The sample preparation involved the incorporation of antimony as the internal standard and dilution using a 0.5% ammonia solution. The analytes were measured at m/z 126.90 for iodide and 120.90 for antimony, respectively. The assay exhibited linearity within the concentration ranges of 0.1–50 ng/mL for iodide and 0.3–100 ng/mL for T4. The methodology demonstrated precision and accuracy, with lower limits of quantification (LLOQs) of 0.1 ng/mL for iodide and 0.3 ng/mL for T4. The inter-day accuracy exceeded 94% for both analytes, and the coefficient of variation (%CV) was below 5%. The method has been effectively employed for dissolution studies of T4 formulations and demonstrates significant potential as a straightforward, accurate, and sensitive analytical methodology for determining T4 concentration in in vitro experiments [13]. 

**Chitravathi et al.(2009)** studied the electrochemical response of Sodium Levothyroxine at a carbon paste electrode using 0.1 M HCl as the supporting electrolyte by cyclic voltammetry. A distinct oxidation peak was seen at 0.78 V, accompanied by subtle and indistinguishable reduction peaks at 0.53 V and 0.32 V. The influence of sodium Levothyroxine concentration and scan rate was examined, revealing that the electrode process is governed by adsorption control. The impact of surfactants such as Sodium Dodecyl Sulphate (SDS), Cetyltrimethylammonium Bromide (CTAB), and Triton X-100 (TX-100) was examined by mobilisation and immobilisation techniques. The concentration effects of all three surfactants were examined. Among these, SDS demonstrated significant augmentation in both oxidation and reduction peak currents [17]. 

**Collier et al.(2011** J.W. Collier et al. (2011) investigated a quick, selective, and sensitive gradient HPLC technique for analysing dissolution samples of levothyroxine sodium tablets. The existing USP approach for levothyroxine (L-T(4)) is insufficient for separating co-elutants from various levothyroxine medicinal product formulations. The USP method for analysing dissolution samples of the pharmaceutical product has demonstrated considerable intra- and inter-day variability. Method variability arises from chromatographic interferences caused by the dissolving media and the formulation excipients. The chromatographic separation of levothyroxine was performed on an Agilent 1100 Series HPLC utilising a Waters Nova-pak column (250 mm × 3.9 mm) with a mobile phase comprising a 0.01 M phosphate buffer (pH 3.0) and methanol (55:45, v/v) in gradient elution, at a flow rate of 1.0 mL/min and a UV detection wavelength of 225 nm. The injection volume was 800 µL, and the column temperature was sustained at 28 °C. The procedure was validated in accordance with USP Category I standards. The validation parameters encompassed accuracy, precision, specificity, linearity, and analytical range. The calibration curve exhibited a linear correlation (r² >0.99) across the analytical range of 0.08–0.8 µg/mL. Accuracy varied from 90 to 110% for low-quality control (QC) standards and from 95 to 105% for medium and high QC standards. Precision remained below 2% across all quality control levels. The approach demonstrated accuracy, precision, selectivity, and linearity for L-T(4) within the analytical range. The HPLC approach was effectively utilised for the analysis of dissolution samples of commercial levothyroxine sodium tablets [8].

## Experimental

### Materials and Solutions

1. Levothyroxine
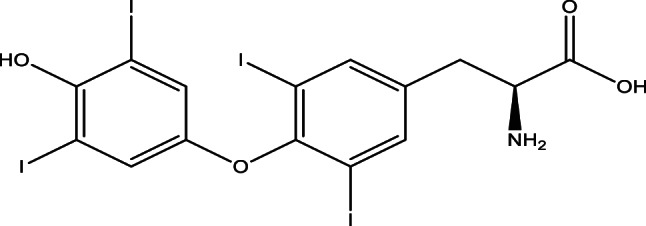

a Formula and Structure: C_15_H_11_I_4_.
b Molecular Weight: 799 g/mol (anhydrous).
c Solubility (sodium salt): Very slightly soluble in water, slightly soluble in alcohol, soluble in dil.alkali solutions.
d Trade name: Eltroxin (100 mcg).
e Manufacturer: ASPEN BAD OLDESLOE GMBH Germany.
f Form : white solid tablets, each tablet contains Levothyroxine sodium equivalent to 100 mcg Levothyroxine as active ingredient.
g Method of preparation: Dissolve specified number of tablets in (1:1) mixture of Ethanol (95%): NaOH (10−4M, pH = 10), filter the solution and keep the filtrate in a refrigerator at temperature below 10° C, to obtain a stock solution of Levothyroxine equals 3.86 × 10−5 mol/L.

2. Ciprofloxacin

a Formula and Structure: C_17_H_18_FN_3_O_3_
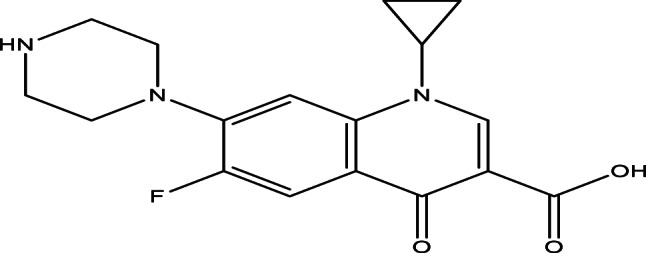
b Molecular weight: 331.346 g/mol (anhydrous).
c Solubility (Hydrochloride form): soluble in water, slightly soluble in Methanol, very slightly soluble in Ethanol.
d Trade name: Ciprofar (750 mg).
e Manufacturer: Pharco.
f Form: almost white or pale yellow solid tablets, each tablet contains Ciprofloxacin Hydrochloride equivalent to 750 mg Ciprofloxacin as active ingredient.
g Method of preparation *(10*^*−4*^*M)*: Dissolve one tablet of Ciprofar in distilled water, stir vigorously and warm the solution if necessary, complete the solution to 100 mL then filter the solution and keep the filtrate. Take 0.45mL of the filtrate and dilute it with distilled water to 100 mL.

3. Alendronic Acid

a Formula and Structure: C_4_H_13_NO_7_P_2_
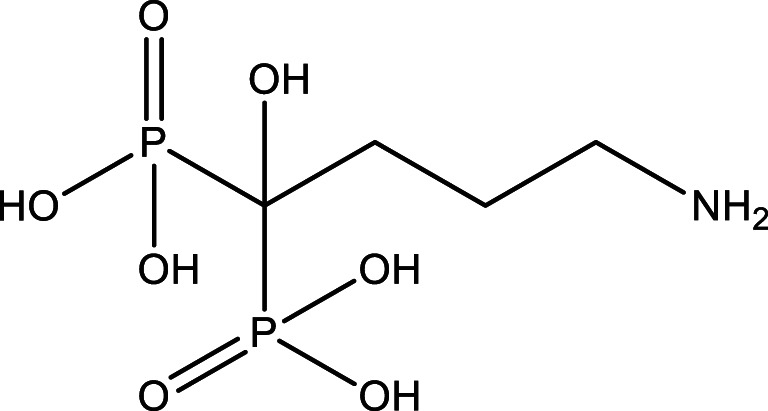
b Molecular weight: 249.097 g/mol (anhydrous). 325.1 g/mol (Sodium Salt Tri Hydrate form)
c Solubility: (sodium salt trihydrate form): soluble in water, very slightly soluble in Methanol, Insoluble in Methylene chloride.
d Trade name: FOSAMAX (70 mg).
e Manufacturer: MSD.
f Form: white solid tablets, each tablet contains Sodium alendronate equivalent to 70 mg Alendronic acid as active ingredient.
g Method of preparation: Dissolve specified quantity of tablets in distilled water to prepare the stock solution with a concentration of 2.80 × 10^−3^mol/L.

4. TrisHClbuffer (pH = 7.4)

a Formula and Structure: C_4_H_11_NO_3_.HC_l_
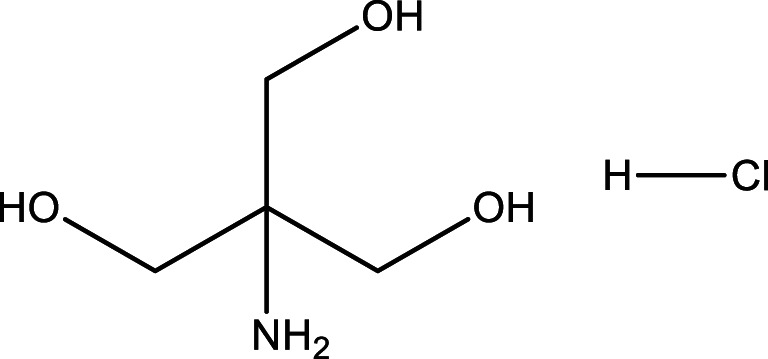
b Molecular weight: 121.14 g/mol (Tris).
c Preparation *(0.05M)*: dissolve3.0285 g of Tris powder in 400 mL of distilled water. Adjust the pH to 7.4 with 0.1 N HCl then complete the volume to 500mL by distilled water.

5. Aluminum Ion Stock Solution

a Formula: Al (NO_3_)_3_·9H_2_O.
b Molecular weight: 375.134 g/mol (nonahydrate).
c Preparation *(10*^*−4*^*M)*: dissolve0.0375 g of Alumium nitrate nonahydrate crystals in distilled water to make 1000mL solution.

6. Ferric Ion Stock Solution

a Formula: Fe (NO_3_)_3_·9H_2_O.
b Molecular weight: 404 g/mol (nonahydrate).
c Preparation *(10*^*−3*^*M)*: dissolve0.404 g of Ferric nitrate crystals in Ethanol to make 100 mL of solution.

7. Calcium Ion Stock Solution

- Formula: CaCl_2_. 2 H_2_O.
- Molecular Weight: 146.98 g/mol.
- Preparation *(10*^*−3*^*M)*:*)*: dissolve0.1469 g of Calcium chloride di-hydrate in distilled water and complete to make 100 mL of solution.

### Instrumentation

Fluorescence measurements were performed using a Thermo Scientific UV-Vis spectrofluorometer equipped with a 1 cm quartz Cuvette. The excitation and emission wavelengths were set at 495 nm and 520 nm, respectively. Slit widths were maintained at 5 nm for both excitation and emission. All experiments were conducted at room temperature (25 ± 2 °C).

### Preparation of Standard Solutions

- A stock solution of Levothyroxine (1 mM) was prepared by dissolving the standard in ethanol with sonication.
- FITC stock solution (100 µM) was prepared in Tris-HCl buffer (pH 7.4).
- Working solutions were freshly prepared by diluting stock solutions with Tris or phosphate buffer.

### Calibration Curve

A series of Levothyroxine solutions (8–64 µmol/L) were prepared by adding appropriate volumes to FITC solution (final FITC concentration: 10 µM). Fluorescence intensity was measured after a 5-minute incubation. A calibration curve was constructed by plotting the quenching (F₀/F) versus Levothyroxine concentration.

### Sample Preparation

#### Pharmaceutical Formulations

Tablets were accurately weighed, powdered, and dissolved in ethanol, followed by filtration and dilution with buffer to the target concentration range.

#### Serum Samples

Blank human serum was spiked with known amounts of Levothyroxine and subjected to simple protein precipitation using acetonitrile (1:1 v/v), followed by centrifugation and dilution with buffer.

### Accuracy and Precision Studies

Accuracy was assessed via recovery experiments at three spiking levels (low, medium, high) in both sample types (*n* = 5). Precision was evaluated by repeatability (intra-day) and intermediate precision (inter-day) analyses, expressed as %RSD.

### Robustness and Ruggedness Studies

Robustness was evaluated by varying buffer pH (± 0.2 units), incubation time (± 1 min), and slight temperature changes (± 2 °C). Ruggedness was assessed by different analysts and on different days.

### Limit of Detection (LOD) and Limit of Quantification (LOQ)

LOD and LOQ were determined based on signal-to-noise ratios of 3:1 and 10:1, respectively.

#### Detection of Levothyroxine Drug Alone

Levothyroxine detection was conducted utilising Fluorescein isothiocyanate isomer I (FITC) as a fluorophore. The measurements were conducted in Tris buffer (pH 7.4) and phosphate buffer (pH 7.4).

The operational solutions comprised 0.10 mL of fluorescein isothiocyanate (FITC) dye stock solution (1.00 × 10^−5^ M) and 2 mL of Tris buffer or phosphate buffer, which were subsequently diluted to 10 mL with distilled water.

The solutions were titrated with several quantities of Levothyroxine solution (1.93 × 10^−4^ M) ranging from 0 to 200 µL to disregard the dilution factor (Table-S1-). All solutions were stimulated at λex = 493 nm, and the emission was recorded at λem = 516 nm.

#### **Detection of Levothyroxine Drug with Interfering Metal Ions (Fe**^**3+**^, **Al**^**3+**^, **Ca**^**2+**^**) and Drugs(Ciprofloxacin**,** Sodium Alendronate)**

The impact of metal ions (Fe^3+^, Al^3+^, Ca^2+^) and pharmaceuticals (Ciprofloxacin, Sodium Alendronate) on the detection of Levothyroxine was assessed using Fluorescein isothiocyanate isomer I (FITC) as a fluorophore. The measurements were conducted in Tris buffer (pH 7.4) and phosphate buffer (pH 7.4).

The working solutions comprised 0.1 mL of FITC dye stock solution (1.00 × 10^−5^ M), 100 µL of Levothyroxine stock solution (1.93 × 10^−4^ M), and 2 mL of Tris buffer or phosphate buffer, which were subsequently diluted to a final volume of 10 mL with distilled water.

The solutions were titrated with varying quantities of interfering metal ions and medication solutions ranging from 0 to 200 µL to disregard the dilution factor (Table S2).

All solutions were stimulated at λex = 493 nm, and the emission was recorded at λem = 516 nm.

#### Temperature Effect on the Interaction of Levothyroxine Drug with FITC Dye

Solutions comprised 0.1 mL of fluorescein isothiocyanate (FITC) dye stock solution (1.0 × 10^−5^ M), 200 µL of levothyroxine stock solution (1.93 × 10^−4^ M), and 2 ml of Tris buffer. The solutions were diluted to 10 mL with distilled water, and emission spectra were recorded at various temperatures (25, 35, 45 °C).

#### Measuring Levothyroxine Hormone (T4) in Human Serum

Known amounts of Levothyroxine in serum sample were recovered by standard addition method. 3.00 mL of human serum was collected from healthy person. The serum sample was left in a refrigerator overnight and centrifuged for 15 min at 8000 rpm 0.2.00 mL of the serum sample was added to solution contained 0.10 mL of (FITC) dye stock solution(1.00 × 10^−5^M), 2.00 mL of Tris buffer and 5.90 mL of distilled water and spiked with known amounts of the standard levothyroxine solution.

## Results and Discussion

### Detection of Levothyroxine Using Fluorescein Isothiocyanate (FITC) Dye

#### Fluorescence Measurements for the Interaction of Levothyroxine with FITC Dye

Levothyroxine possesses three ionisable groups: a carboxy group (pKa1 = 2.40), a phenolic group (pKa2 = 6.87), and an amino group (pKa3 = 9.96), allowing it to exist as a cation, zwitterion, or anion. The aqueous solubility of Levothyroxine diminishes when pH rises from 1 to 3 and thereafter increases above pH 7 [28]– [29].

Fluorescein isothiocyanate (FITC) exhibits a yellow-orange hue and has an absorption maximum at 493 nm. Upon excitation, it exhibits a yellow-green hue with an emission peak at 525 nm.

It serves as a fluorescent labelling agent for proteins, a fluorescent tracer for protein detection, and a reagent in the fluorescent antibody method for the swift identification of infections. The isothiocyanate group interacts with amino terminal and primary amines in proteins. It is utilised as a reagent for the micro-sequencing of proteins and peptides by HPLC.

The interaction of varying doses of Levothyroxine with 10^−7^ M of Fluorescein isothiocyanate (FITC) dye in Tris buffer (pH 7.4) and phosphate buffer (pH 7.4), with excitation at λex = 493 nm and emission seen at 516 nm, is illustrated in Figures (–S1-, –S2-).

Figures S1 and S2 illustrate the fluorescence quenching behavior of Fluorescein isothiocyanate (FITC) dye upon incremental addition of Levothyroxine under controlled experimental conditions. Specifically, a constant concentration of FITC dye (1.0 × 10⁻⁷ M) was used in both Tris buffer (pH 7.4) and phosphate buffer (pH 7.4), with fluorescence measurements performed at an excitation wavelength of λ_ex_ = 493 nm and emission monitored at λ_em_ = 516 nm.

In Figure S1, the fluorescence emission spectra of FITC were recorded after the sequential addition of varying doses of Levothyroxine in Tris buffer. The results clearly demonstrate a gradual and systematic decrease in the fluorescence intensity of FITC with increasing Levothyroxine concentration. This quenching behavior suggests a specific interaction between Levothyroxine and FITC, likely involving either static or dynamic quenching mechanisms such as complex formation or collisional deactivation.

Similarly, Figure S2 presents the corresponding fluorescence response in phosphate buffer under identical experimental settings. A comparable quenching trend is observed, indicating that the interaction between Levothyroxine and FITC is consistent across both buffer systems. The use of two different buffers allows assessment of the influence of buffer composition and ionic strength on the binding behavior and fluorescence quenching process.

These fluorescence studies form the foundational step in evaluating the feasibility of FITC as a selective fluorescent probe for Levothyroxine detection. They also provide the necessary basis for subsequent quantitative analysis, including the determination of quenching constants and binding parameters through Stern–Volmer and other kinetic models, as further detailed in the following sections and supplementary figures.

Figures S3 and S4 present the calibration curves established for the quantitative detection of Levothyroxine using the fluorescence quenching response of FITC dye in two distinct buffer systems. These calibration plots serve as a crucial part of the study by providing a quantitative relationship between Levothyroxine concentration and the corresponding decrease in fluorescence intensity.

In Figure S3, the calibration curve was generated using FITC dye in Tris buffer (pH 7.4). The plot demonstrates a clear and linear relationship between the increasing concentration of Levothyroxine and the corresponding quenching of fluorescence intensity, indicating the reliability of FITC as a fluorescent sensor under these conditions. Based on the standard method for calculating detection limits, the limit of detection (LOD) was determined using the equation LOD = 3σ/slope, where σ represents the standard deviation of the blank measurements and the slope is derived from the linear regression of the calibration plot. Accordingly, the LOD for Levothyroxine detection in Tris buffer was calculated to be **9.60 × 10⁻⁶ mol/L**, indicating a satisfactory sensitivity level for the probe under the experimental conditions.

Similarly, Figure S4 depicts the calibration plot for Levothyroxine detection in phosphate buffer (pH 7.4) using FITC dye. A consistent linear quenching behavior is observed, confirming that the fluorescence response of FITC remains stable and predictable in different buffer environments. The LOD calculated from this calibration plot was found to be **8.80 × 10⁻⁶ mol/L**, suggesting slightly enhanced sensitivity in phosphate buffer compared to Tris buffer.

These calibration studies collectively validate the effectiveness of FITC dye as a fluorescence-based probe for the quantitative detection of Levothyroxine in aqueous media. The demonstrated low detection limits in both buffer systems highlight the method’s potential for practical applications in pharmaceutical analysis and bioanalytical sensing, while also demonstrating the robustness of the method across different buffer conditions.

Figures S5 and S6 present the Stern–Volmer plots derived from the fluorescence quenching data of FITC dye by Levothyroxine in Tris buffer and phosphate buffer, respectively. These plots are essential for quantitatively evaluating the quenching mechanism and for determining the quenching constants (Ksv) which provide insight into the quenching efficiency.

The Stern–Volmer equation, F0/F = 1 + Ksv[Q], where F₀ and F represent the fluorescence intensities of FITC in the absence and presence of the quencher (Levothyroxine), respectively, and [Q] is the quencher concentration, was applied to the experimental data. The resulting plots show a linear relationship between F₀/F and Levothyroxine concentration, indicating that the quenching process follows a **dynamic quenching mechanism** under the studied conditions.

In Figure S5, the Stern–Volmer plot for the system in **Tris buffer (pH 7.4)** demonstrates a strong linear trend with a calculated quenching constant (Ksv​) of **6.92 × 10² L/mol**, reflecting a high quenching efficiency in this buffer system. Similarly, Figure S6 shows the corresponding Stern–Volmer plot in **phosphate buffer (pH 7.4)**, where the calculated Ksv​ value was slightly lower, at **5.06 × 10² L/mol**, yet still within the range indicative of efficient fluorescence quenching.

The difference in quenching constants between the two buffer systems can be attributed to variations in buffer composition, ionic strength, or subtle changes in dye–quencher interactions. However, in both cases, the high linearity and significant Ksv​ values clearly confirm that Levothyroxine acts as an effective quencher for FITC fluorescence under physiological pH conditions.

These findings not only support the suitability of FITC as a reliable fluorescence probe for Levothyroxine detection but also provide quantitative parameters that further validate the interaction mechanism proposed in this study.

##### Binding Constant Determination Using Lineweaver–Burk Plots

Figures S7 and S8 depict the Lineweaver–Burk plots derived from the fluorescence quenching experiments of FITC dye with Levothyroxine in Tris buffer and phosphate buffer, respectively. These plots were constructed to accurately determine the binding constant (Ka) of Levothyroxine toward FITC under the applied experimental conditions.

The analysis is based on the modified Stern–Volmer approach, where the reciprocal of fluorescence change is plotted against the reciprocal of quencher concentration according to the Lineweaver–Burk equation:

$$\:\frac1{F0-F}=\frac1{Ka(Fmax-F0)\left[Q\right]}+\frac1{F_0\:-\:Fmax}\:$$

Here, F₀​ and F represent the fluorescence intensities of FITC in the absence and presence of Levothyroxine, respectively, and [Q] is the quencher (Levothyroxine) concentration. This linearization allows for the precise calculation of the binding constant from the slope and intercept of the plot.

In Figure S7, the Lineweaver–Burk plot for the interaction in **Tris buffer (pH 7.4)** reveals a strong linear relationship, yielding a calculated binding constant of **3.33 × 10⁴ L/mol**, indicating a high binding affinity between FITC and Levothyroxine in this buffer system. This suggests strong molecular interactions under the experimental conditions employed.

In Figure S8, the corresponding plot for **phosphate buffer (pH 7.4)** also shows a linear trend but with a lower binding constant of **3.90 × 10³ L/mol**, reflecting a moderate interaction strength in this environment. The decrease in binding affinity in phosphate buffer compared to Tris buffer may be attributed to differences in ionic strength, buffer composition, or competitive interactions with buffer components.

These results provide quantitative evidence for the interaction between FITC and Levothyroxine and further confirm the feasibility of using FITC as a fluorescence-based probe for sensitive Levothyroxine detection in biological and pharmaceutical samples. Additionally, they highlight the importance of buffer selection in optimizing sensing performance.

The analytical validation parameters for detecting Levothyroxine using Fluorescein isothiocyanate (FITC) in Tris and phosphate buffer are presented in Table 2.

**Table 2:** Method analytical validation parameters

Parameter	Tris buffer	Phosphate buffer	
Linear range, mol/L	8.00 × 10^−6^ − 6.40 × 10^−5^	8.00 × 10^−6^ − 6.40 × 10^−5^	
Limit of detection(LOD), mol/L	9.60 × 10^−6^	8.80 × 10^−6^	
Limit of quantitation (LOQ), mol/L	3.20 × 10^−5^	2.94 × 10^−5^	
Regression equation, Y^a^	Y=−8.23*10^5^ (x) + 815.5	Y=−9.48*10^5^ (x) + 958.5	
Intercept (a)	815.5	958.5	
Slope(b)	−8.23×10^5^	−9.48×10^5^	
Standard deviation (σ)	2.63438	2.78379	
Regression coefficient (r^2^)	0.96	0.97	

#### Interfering species in the determination of Levothyroxine using FITC dye

In this study, we evaluated the assay’s selectivity by testing potential interferents relevant to the method’s intended applications in pharmaceutical quality control and high-dose serum analysis. Specifically, we investigated:

- **Ciprofloxacin** and **Alendronate**—two commonly co-administered medications known to interact with Levothyroxine pharmacokinetically and affect its absorption and bioavailability.
- **Metal ions** such as Fe³⁺, Al³⁺, and Ca²⁺, which are frequently encountered in pharmaceutical formulations, dietary supplements, and clinical settings, and are known to complex with Levothyroxin.

The rationale for selecting these species is based on their **direct relevance to pharmaceutical and therapeutic contexts** where this assay would be most applicable. Our results demonstrated that while these compounds exhibited varying degrees of fluorescence quenching, the assay maintained acceptable performance within the tested concentration ranges. 

We acknowledge that clinical samples may contain additional endogenous interferents (e.g., proteins, fatty acids, and bilirubin). However, the serum recovery studies presented in Sect. 3.4, using untreated diluted human serum, showed acceptable recoveries and minimal matrix interference under our testing conditions, supporting the method’s feasibility for high-dose serum applications.

Nonetheless, we recommend that future work expand the interference panel to include additional biological components to evaluate potential applications in more complex clinical matrices.

The effect of addition of Ciprofloxacin, Sodium alendronate, Al^3+^,Fe^3+^, Ca^2+^to the solution containing the fluorescent dye in a concentration of 1.00 × 10^−6^ mol/L keeping the levothyroxine concentration at 3.20 × 10^−5^ mol/L. The variation of the fluorescence intensity with addition of different concentrations of the studied interfering species was measured until a change in the initial fluorescence intensity was observed to be 10% tolerance effect as shown in figures (-S9- to -S13-). It is clearly observed that all examined species exhibit quenching effect of the initial fluorescence for the solution containing FITC dye and levothyroxine. Table –S3- illustrates the tolerance value, where as shown the most species affecting the determination of Levothyroxine using FITC dye are Al^3+^, Fe^3+^, Ca^2+^ rather than Ciprofloxacin or Sodium alendronate.

#### Binding mode of Levothyroxine using FITC dye

The molecular forces contributing to the interaction between the two studied substances may be Van’der Waals interaction, hydrogen bond, ionic, electrostatic and hydrophobic interactions. The signs and magnitude of thermodynamic parameters can be accounted for the main forces contributing interactions.

If the enthalpy change doesn’t vary greatly over the temperature range studied, then its value and that of enthalpy change can be determined from the Van’t Hoff equation:

2 $$\mathrm{lnK}=\frac{-\triangle H^\circ}{RT}+\frac{\triangle S^\circ}R$$

Where

3 $$\triangle G^\circ=\triangle H-T\triangle S^\circ$$

##### Temperature-Dependent Binding Analysis

To further investigate the thermodynamic aspects of the interaction between Levothyroxine and FITC dye, fluorescence quenching experiments were performed at three different temperatures (25 °C, 35 °C, and 45 °C). The corresponding fluorescence spectra are presented in Figure S-14

The results clearly show an increase in fluorescence quenching with rising temperature, indicating that the quenching process is enhanced at elevated temperatures. This temperature-dependent behavior suggests that the binding between Levothyroxine and FITC is likely governed by a **dynamic quenching mechanism** associated with diffusion-controlled interactions.

**Fig. 4:**
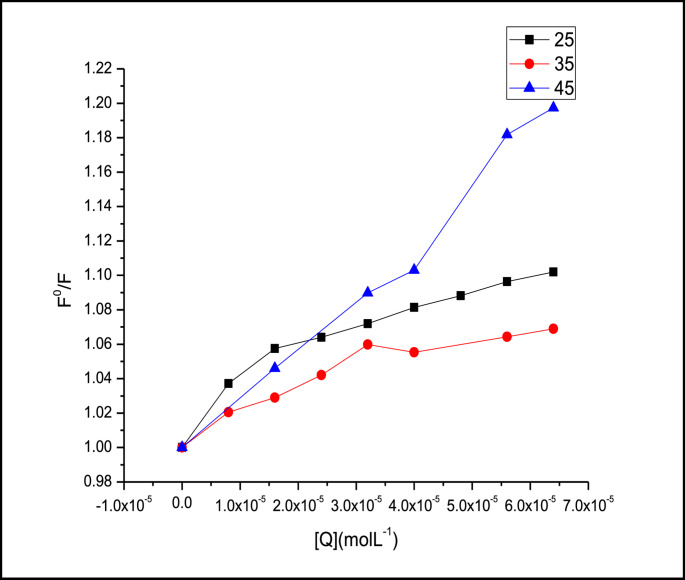
Stern-Volmer plot for the interaction of Levothyroxine with FITC at different temperatures (**a**) 250 C (**b**) 350 C (**c**) 450 C

**Fig. 5:**
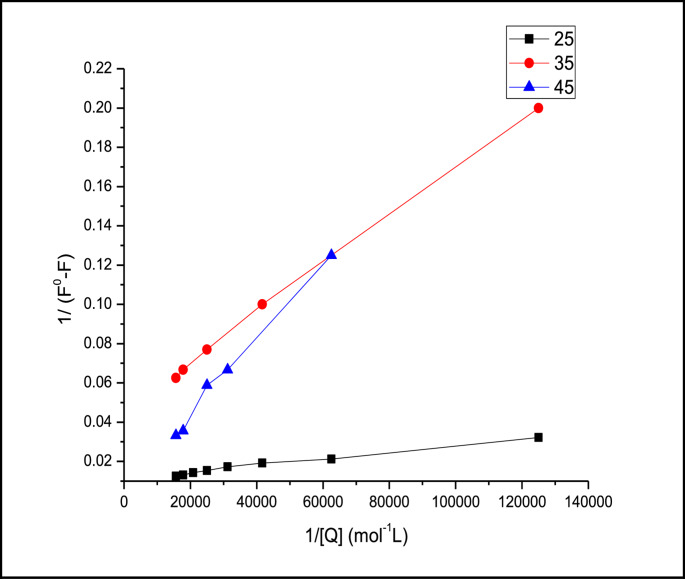
Lineweaver-Burck plot for the interaction of Levothyroxine with FITC at different temperatures (**a**) 250 C (**b**) 350 C (**c**) 450 C

These findings are consistent with the thermodynamic parameters calculated from the main quenching data (Figs. 3–5), where positive enthalpy changes were observed, confirming an endothermic binding process. The temperature-dependent increase in quenching further supports the hypothesis that hydrophobic forces and non-covalent interactions play a significant role in the binding mode of Levothyroxine to FITC under the studied conditions. 

The quenching data were fitted in Stern-Volmer equation at each temperature (Fig. 4), the biomolecular quenching constants were found to be equal 1.38 × 10^3^, 1.07 × 10^3^ and 3.14 × 10^3^ mol^−1^L at 25,35 and 45 °C respectively. The fluorescence intensity of FITC decreased linearly with increasing concentrations of levothyroxine, yielding Stern–Volmer plots with correlation coefficients. Furthermore, the Stern–Volmer quenching constant was observed to increase with temperature (Table 2), indicating enhanced quenching efficiency at higher thermal energy. This pattern is consistent with a **dynamic (collisional) quenching mechanism**, where diffusion plays a central role. hence the mechanism could be attributed to dynamic or collosional one.

Using Lineweaver-Burck equation, the binding constants were calculated (Fig. 5) and the values were found to be 3.33 × 10^4^, 3.26 × 10^3^ and 2.82 × 10^3^ mol^−1^L at 25,35 and 45 °C respectively.

To further understand the thermodynamic features of this interaction, the van’t Hoff plot of ln K versus 1/T was constructed (Fig. 5). The linear relationship observed allowed us to estimate the enthalpy change (ΔH°) and entropy change (ΔS°) associated with the process.

**Fig. 6:**
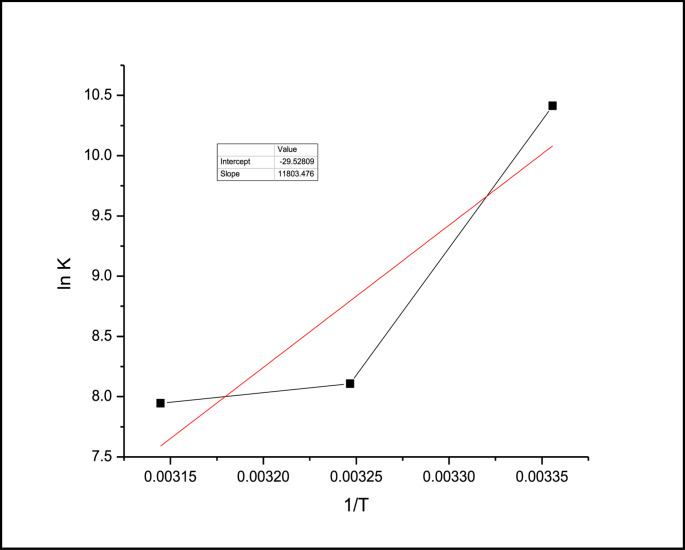
Ln K vs. 1/T for Levothyroxine-FITC system

The calculated thermodynamic parameters indicate that the process is endothermic (positive ΔH°) and accompanied by a positive ΔS°, consistent with a diffusion-controlled, dynamic quenching mechanism rather than static complex formation. The positive entropy change suggests increased disorder, typical of dynamic collisions and solvation changes during the quenching interaction. (Fig. 6)

The values of ΔH°,ΔS° and ΔG° are summarized in table –S4-

The negative values of ΔG° reveal that the interaction process is spontaneous. From the negative values of and the positive values of electrostatic interaction could be the nature of force between the two molecules.

#### Detection of Levothyroxine in Blood Serum Using Fluoresceinisothiocyanate (FITC) Dye

Known amounts of Levothyroxine in serum sample were recovered by standard addition method. Three milliliters of human serum was collected from healthy person. The serum sample was left in a refrigerator overnight and centrifuged for 15 min at 8000 rpm. Two milliliters of the serum sample was spiked with known amounts of the standard Levothyroxine solution. The added concentration of the drug was determined using FITC dye in the presence of Tris-HCl buffer (pH = 7.4). The recoveries were in the range of 102.02 to 103.93% in the serum sample confirming minimal matrix interference. Standard addition curves (R^2^ > 0.98) demonstrated robustness in biological samples. This performance aligns with clinical requirements, positioning the method as a viable alternative to immunoassays for serum T4 monitoring. The results are summarized in Table 3.

**Table 3:** Recovery of Levothyroxine in serum sample using standard addition method

Added mol/L	Observed mol/L	Observed mol/L
9.65 × 10^−7^	1.63 × 10^−5^	102.02 ± 1.4
1.93 × 10^−6^	3.27 × 10^−5^	102.26 ± 1.2
2.90 × 10^−6^	4.98 × 10^−5^	103.93 ± 1.6

The results of a recovery study also performed under the standard addition method indicated that the method can be applied to assess Levothyroxine in serum samples with satisfactory results.

#### Comparison with Various Methods for Detection of Levothyroxine

Table 4 illustrates comparison of the analytical parameters where the previous methods for the determination of levothyroxine, where the previous methods have a high requirement of instrumental precision and the analysis method is also very complicated. Our method is fast, more economic and also exhibit the ability to detect a relatively low concentration [12].

**Table 4:** Comparison of the present work with the previous analytical methods for the determination of Levothyroxine

Method	LOD	Linear range	Reference
Reversed-phase type HPLC	**<** 0.13µmol/L	0.64–643.60µmol/L	H.Gika et al(2004)
HPLC	**-**	0.10–1.30 µmol/L	J.W.Collier et al.(2011)
kinetic method (based on the catalytic effect of thyroxine on the oxidation of As(III) by Mn(III) metaphosphate)	2.70 nmol/L	7.0–30.0 nmol/L	F.pastor et al(2007)
FITC probe	9.60µmol/L	8.00- 64.00 µmol/L	This work

#### Greenness and Blueness Evaluation of the Method

In alignment with contemporary principles of sustainable analytical chemistry, the greenness of the proposed spectrofluorometric method for levothyroxine determination was evaluated using the AGREE (Analytical GREEnness) metric. This tool provides a comprehensive assessment of the method’s environmental impact by examining factors such as solvent and reagent safety, energy consumption, waste generation, and occupational hazards.

The method exhibited a favorable AGREE score of 0.69 (see Table S5), as illustrated graphically in Figure S15. This high score reflects the method’s environmentally benign nature—most notably due to the use of aqueous buffer systems and the complete avoidance of hazardous organic solvents. These results confirm the method’s green profile, placing it on par with or exceeding other recent green spectrofluorometric strategies reported in the literature for pharmaceutical analysis [30–33].

These evaluations confirm that the proposed method is not only analytically effective and sensitive, but also environmentally sustainable and socially responsible, aligning well with current principles of green and blue analytical chemistry [30–37].

## Conclusion

In this study, we developed and validated a rapid, simple, and cost-effective spectrofluorometric method for the determination of Levothyroxine (T₄) based on static fluorescence quenching of fluorescein isothiocyanate (FITC). The assay operates at physiological pH without requiring derivatization, hazardous chemicals, or complex sample preparation. With a total assay time of less than six minutes and a detection limit of 9.6 µmol L⁻¹ (equivalent to ~ 7.7 µg L⁻¹), the method is well suited for applications where speed, ease of use, and low cost are critical—such as quality control in pharmaceutical formulations and routine monitoring of elevated serum T₄ levels.

While the method does not match the ultra-trace sensitivity of LC-MS/MS or ICP-MS techniques, it offers distinct advantages in terms of throughput, instrument simplicity, and reagent accessibility. While not intended for nanomolar-level detection of free T₄, the method meets analytical demands in contexts where total levothyroxine levels are elevated or intentionally high—as in dosage verification, tablet formulation control, and overdose response. The assay shows strong selectivity, minimal interference from common co-medications, and acceptable agreement with published therapeutic ranges. As such, this approach fills a practical gap in current analytical strategies, offering a viable alternative for laboratories operating under resource constraints or high-throughput screening conditions. Future studies are warranted to assess potential interferences from additional endogenous biological components—such as bilirubin, lipids, and serum proteins—to further validate the assay’s applicability to complex clinical matrices beyond the pharmaceutical and high-dose serum contexts addressed here.

## Supplementary Information

Below is the link to the electronic supplementary material.

ESM 1 (DOCX 172 KB)

ESM 2 (DOCX 18.0 KB)

## Data Availability

No datasets were generated or analysed during the current study.
