# Correction: A Rapid Fluorescence Quenching Assay for Total Levothyroxine Quantification in Pharmaceutical and Supratherapeutic Serum Samples

**DOI:** 10.1007/s10895-025-04640-0

**Published:** 2025-11-25

**Authors:** Enas T. Abdel-Salam, Zeinab M. Anwar, Ahmed Z. Ibrahim, Hend M. Musatfa

**Affiliations:** 1 Faculty of Science, Chemistry Department, Suez Canal University, Ismailia, Egypt; 2 Research Faculty of Science, Chemistry Department, El- Arish University, ElArish, Egypt

**Correction: Journal of Fluorescence**

10.1007/s10895-025-04510-9.

In this article, the first sentence under the "Instrumentation" section was incorrectly given as "Fluorescence measurements were performed using a [model, brand] spectrofluorometer equipped with a 1 cm quartz Cuvette" but should have been "Fluorescence measurements were performed using a Thermo Scientific UV-Vis spectrophotometer equipped with a 1 cm quartz Cuvette". The original article has been corrected.

