# Supplementary material for: A Rapid Fluorescence Quenching Assay for Total Levothyroxine Quantification in Pharmaceutical and Supratherapeutic Serum Samples
Source: J Fluoresc. 2025 Sep 15;35(12):13619–34. doi: 10.1007/s10895-025-04510-9 (PMC12858594; doi:10.1007/s10895-025-04510-9)
Supplement: Supplementary file 1 — (DOCX 172 KB) [file 10895_2025_4510_MOESM1_ESM.docx]

Figure –S1-. Fluorescence emission spectra of FITC (1.0 × 10⁻⁷ mol/L) in Tris buffer (pH 7.4) at 25 °C, excited at 493 nm and monitored at 516 nm, upon incremental addition of Levothyroxine (0–1.0 × 10⁻⁴ mol/L). The blank spectrum and quenched spectra are shown.

Figure –S2-. Fluorescence emission spectra of FITC (1.0 × 10⁻⁷ mol/L) in phosphate buffer (pH 7.4), showing emission intensity decrease with increasing Levothyroxine concentrations.

Figure –S3-. Calibration curve of Levothyroxine in Tris buffer using FITC dye, with LOD determined as 9.60 × 10⁻⁶ mol/L based on 3σ/slope.

Figure –S4-. Calibration curve of Levothyroxine in phosphate buffer using FITC dye, with LOD determined as 8.80 × 10⁻⁶ mol/L.

Figure –S5- Stern-Volmer plot of FITC quenching by Levothyroxine in Tris buffer, indicating dynamic quenching with Ksv = 6.92 × 10² mol⁻¹ L.

Figure –S6- Stern-Volmer plot of FITC quenching by Levothyroxine in phosphate buffer, indicating dynamic quenching with Ksv = 5.06 × 10² mol⁻¹ L.

Figure –S7- Lineweaver-Burk plot showing binding of Levothyroxine to FITC dye in Tris buffer with a binding constant of 3.33 × 10⁴ mol⁻¹ L.

Figure –S8- Lineweaver-Burk plot showing binding of Levothyroxine to FITC dye in phosphate buffer with a binding constant of 3.90 × 10³ mol⁻¹ L.

| ****  Fig–S9- Sodium Alendronate interference effect |
| --- |
| ****  Fig –S10- Al^3+^ interference effect |
| ****  Fig –S11- Ca^2+^ interference effect |
| ****  Fig–S12- Ciprofloxacin interference effect |
| ****  Fig–S13- Fe^3+^ interference effect |

| (a)   | |
| --- | --- |
| (b)   | (c)   |

Figure –S14- Effect of Levothyroxine concentration on the fluorescence of FITC dye at different temperatures.

1. 25^0^ C (b) 35^0^ C (c) 45^0^ C


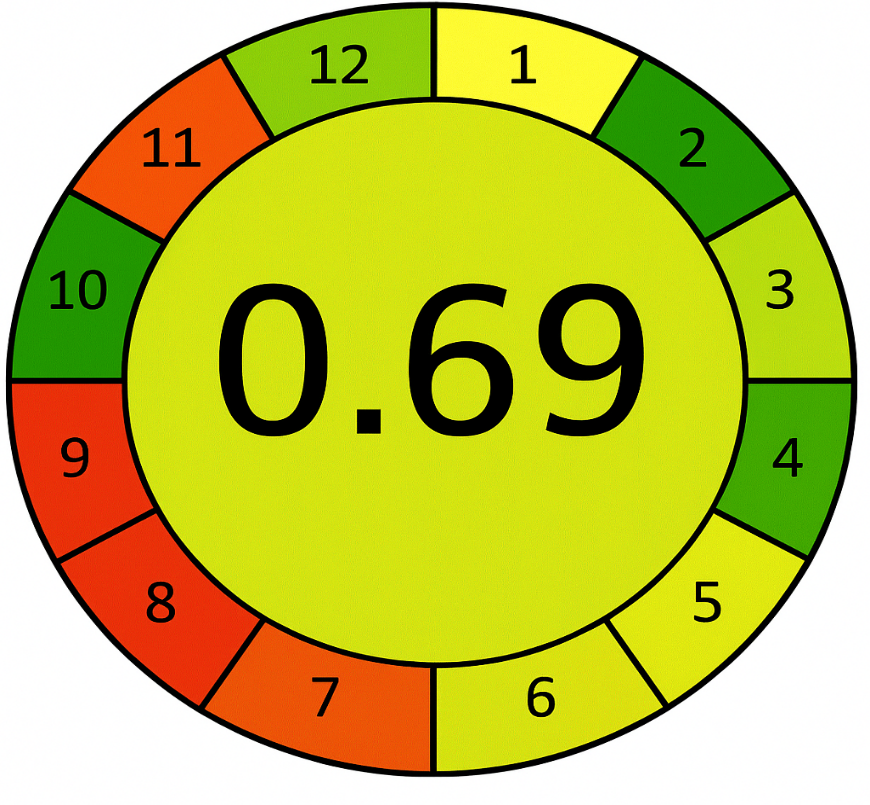


Figure –S15- AGREE diagram (Analytical GREEnness metric) representing the greenness profile of the proposed spectrofluorometric method for levothyroxine detection. Each of the 12 segments corresponds to one of the 12 principles of green analytical chemistry, with color coding from green (favorable) to red (unfavorable). The overall AGREE score was calculated to be **0.69**, indicating good compliance with green analytical practices.
