# Supplementary material for: A Rapid Fluorescence Quenching Assay for Total Levothyroxine Quantification in Pharmaceutical and Supratherapeutic Serum Samples
Source: J Fluoresc. 2025 Sep 15;35(12):13619–34. doi: 10.1007/s10895-025-04510-9 (PMC12858594; doi:10.1007/s10895-025-04510-9)
Supplement: Supplementary file 2 — (DOCX 18.0 KB) [file 10895_2025_4510_MOESM2_ESM.docx]

Table-S1-Effect of Levothyroxine concentrations on fluorescence spectra of FITC dye in TrisHCl buffer (pH=7.4) and phosphate buffer (pH=7.4)

| **FITC dye** | | **Levothyroxine** | | **Distilled water** | **Buffer** |
| --- | --- | --- | --- | --- | --- |
| Volume(ml) | Concentration(M) | Volume(µl) | Concentration(M) | Volume(ml) | Volume(ml) |
| 0.1 | 1.00x10^-7^ | **0.0** | 0.0 | **8** | **2** |
| 0.1 | 1.00x10^-7^ | **25** | 4.83x10^-7^ | **8** | **2** |
| 0.1 | 1.00x10^-7^ | **50** | 9.65x10^-7^ | **8** | **2** |
| 0.1 | 1.00x10^-7^ | **75** | 1.45x10^-6^ | **8** | **2** |
| 0.1 | 1.00x10^-7^ | **100** | 1.93x10^-6^ | **8** | **2** |
| 0.1 | 1.00x10^-7^ | **125** | 2.41x10^-6^ | **8** | **2** |
| 0.1 | 1.00x10^-7^ | **150** | 2.90x10^-6^ | **8** | **2** |
| 0.1 | 1.00x10^-7^ | **175** | 3.38x10^-6^ | **8** | **2** |
| 0.1 | 1.00x10^-7^ | **200** | 3.86x10^-6^ | **8** | **2** |

Table-S2-Effect of interfering agents concentrations on fluorescence spectra of FITC dye bonded with Levothyroxine in TrisHCl buffer (pH=7.4)

| **FITC dye** | | **Interfering agent** | | **Distilled water** | **L-thyroxine** | **Buffer** |
| --- | --- | --- | --- | --- | --- | --- |
| Volume(ml) | Concentration(M) | Volume(µl) | Concentration(M) | Volume(ml) | Volume(µl)) | Volume(ml) |
| 0.1 | 1.00x10^-7^ | **0.0** | 0.0 | **7** | **100** | **2** |
| 0.1 | 1.00x10^-7^ | **25** | 8.00x10^-6^ | **7** | **100** | **2** |
| 0.1 | 1.00x10^-7^ | **50** | 1.60x10^-5^ | **7** | **100** | **2** |
| 0.1 | 1.00x10^-7^ | **75** | 2.40x10^-5^ | **7** | **100** | **2** |
| 0.1 | 1.00x10^-7^ | **100** | 3.20x10^-5^ | **7** | **100** | **2** |
| 0.1 | 1.00x10^-7^ | **125** | 4.00x10^-5^ | **7** | **100** | **2** |
| 0.1 | 1.00x10^-7^ | **150** | 4.80x10^-5^ | **7** | **100** | **2** |
| 0.1 | 1.00x10^-7^ | **175** | 5.60x10^-5^ | **7** | **100** | **2** |
| 0.1 | 1.00x10^-7^ | **200** | 6.40x10^-5^ | **7** | **100** | **2** |

Table-S3-Tolerance of Levothyroxine using fluorescein isothiocyanate(FITC) in the presence of different interfering species.

| **Interfering species** | **Concentration coexisting** | |
| --- | --- | --- |
|  | **µM** | **µg/ml** |
| **Ciprofloxacin** | 255 | 84.49 |
| **Sodium alendronate** | 235 | 58.53 |
| **Fe^3+^** | 218 | 5.88 |
| **Ca^2+^** | 215 | 12.04 |
| **Al^3^**^+^ | 310 | 12.4 |

Table-S4-Thermodynamic and binding parameters for the interaction of Levothyroxine with FITC.

| Temperature (K) | K_sv_  (mol^-1^L) | K  (mol^-1^L) | $\Delta G^{\circ}$  (KJmol^-1^) | $\Delta H^{\circ}$  (KJmol^-1^) | $\Delta S^{\circ}$  (Jmol^-1^K^-1^) |
| --- | --- | --- | --- | --- | --- |
| 298 | 1.38 x10^3^ | 3.33 X 10^3^ | -25.76 | -11.80 | +46.8 |
| 303 | 1.07 x10^3^ | 3.26 X 10^3^ | -20.73 |  | +28.98 |
| 318 | 3.14 x10^3^ | 2.82 X 10^3^ | -20.97 |  | +28.83 |
